# Incidental Colonic Pneumatosis Intestinalis With Pneumoperitoneum Managed Conservatively

**DOI:** 10.1002/ccr3.73221

**Published:** 2026-07-25

**Authors:** Takuya Omura, Tsukasa Aritake

**Affiliations:** ^1^ Department of Metabolic Research, Research Institute National Center for Geriatrics and Gerontology Obu Japan; ^2^ Department of Diabetes and Endocrinology, Hospital National Center for Geriatrics and Gerontology Obu Japan; ^3^ Department of Surgery, Hospital National Center for Geriatrics and Gerontology Obu Japan; ^4^ Department of Surgery Nagoya University Graduate School of Medicine Nagoya Japan

**Keywords:** conservative management, diabetes mellitus, pneumatosis intestinalis, pneumoperitoneum

## Abstract

Pneumoperitoneum accompanying pneumatosis intestinalis can mimic intestinal perforation but does not always require surgery. In asymptomatic, hemodynamically stable patients without high‐risk CT features, conservative management with careful observation and medication review may help avoid unnecessary laparotomy.

## 1 Clinical Question

In pneumatosis intestinalis, when does pneumoperitoneum warrant emergency surgery rather than conservative management?

## 2 Answer

Pneumoperitoneum in pneumatosis intestinalis does not automatically indicate intestinal perforation. Careful observation may be appropriate in clinically stable patients without abdominal symptoms or high‐risk CT features and, when laboratory data are available, without laboratory findings strongly suggestive of bowel ischemia.

## 3 Case Summary

A 74‐year‐old man was referred to the surgical department after pneumatosis intestinalis was incidentally identified on computed tomography (CT). He had undergone contrast‐enhanced CT of the chest to evaluate chronic cough, and findings suggestive of pneumatosis intestinalis were noted on the inferior slices that included the upper abdomen. He denied abdominal pain, distension, nausea, vomiting, diarrhea, hematochezia, or any other abdominal symptoms.

He had a 19‐year history of type 2 diabetes mellitus and had been treated with imeglimin, dulaglutide, and insulin degludec/aspart. His medical history also included interstitial pneumonia, rheumatoid arthritis, hypertension, chronic kidney disease, and paroxysmal atrial fibrillation. On presentation, he was afebrile and hemodynamically stable. The abdomen was soft and non‐distended, without tenderness or peritoneal signs. Laboratory testing showed mild leukocytosis, and the C‐reactive protein level was not elevated.

Subsequent contrast‐enhanced CT demonstrated prominent intramural gas predominantly involving the ascending colon, consistent with pneumatosis intestinalis, with associated pneumoperitoneum and retroperitoneal air (Figure 1). Bowel wall enhancement was preserved. There was no portal venous gas, ascites, or other CT findings suggestive of intestinal ischemia, despite the presence of pneumoperitoneum. Given the absence of abdominal symptoms, stable vital signs, preserved enhancement, and lack of high‐risk CT features, conservative management was selected.

**FIGURE 1:**
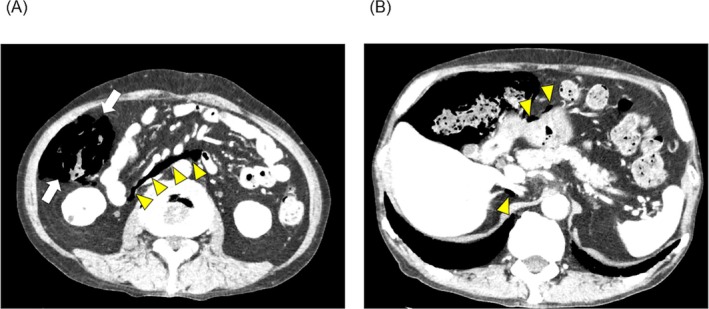
Pneumatosis intestinalis with associated pneumoperitoneum and retroperitoneal air. (A) Contrast‐enhanced CT shows prominent intramural gas in the ascending colon, consistent with pneumatosis intestinalis (arrows) and retroperitoneal air (arrowheads). (B) Contrast‐enhanced CT shows intraperitoneal free air (arrowheads).

The patient was managed with temporary fasting only, without antibiotics or supplemental oxygen, and remained clinically stable. During hospitalization, his diabetes regimen was simplified to insulin degludec/aspart alone, and the other antidiabetic agents were withheld. Follow‐up CT demonstrated decreased intramural gas in the ascending colon, indicating interval improvement of pneumatosis intestinalis (Figure 2), and he was discharged without surgical intervention. At outpatient follow‐up, imeglimin and dulaglutide had not been restarted after an individualized review of his treatment regimen.

**FIGURE 2:**
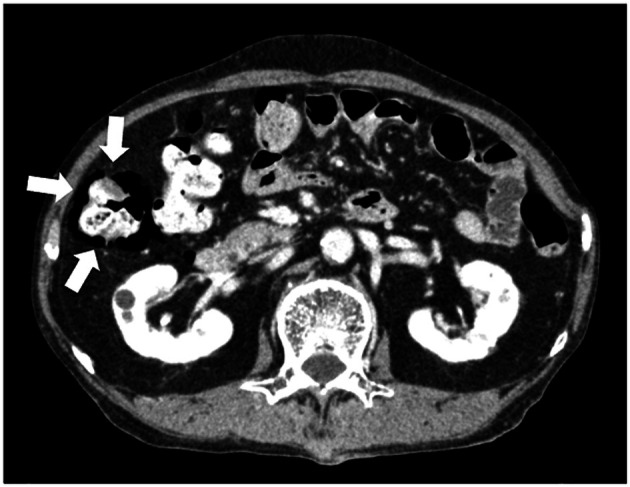
Follow‐up CT shows decreased intramural gas in the ascending colon, indicating interval improvement of pneumatosis intestinalis (arrows).

Pneumatosis intestinalis is a radiologic finding with a broad clinical spectrum, ranging from benign incidental disease to life‐threatening intestinal ischemia [1]. Even when imaging findings are striking, management should be guided by the overall clinical picture and accompanying CT features [2]. A structured approach that integrates symptoms, laboratory data, and CT findings can help avoid unnecessary laparotomy in benign cases while identifying patients who require urgent intervention [3].

In patients with diabetes mellitus, gastrointestinal dysmotility related to autonomic neuropathy is well described and may predispose susceptible individuals to gas‐related phenomena through altered motility and increased intraluminal pressure. In addition, glucagon‐like peptide‐1 receptor agonists are known to slow gastric emptying and affect gastrointestinal motility. However, their relationship to pneumatosis intestinalis remains unclear. This case underscores the importance of careful risk stratification and medication review when pneumatosis intestinalis is identified incidentally.

## Author Contributions

**Takuya Omura:** conceptualization, investigation, supervision, writing – review and editing. **Tsukasa Aritake:** conceptualization, investigation, writing – original draft, writing – review and editing.

## Funding

This study was supported by JSPS KAKENHI (JP23K16812) and Research Funding for Longevity Science (24‐1) from the National Center for Geriatrics and Gerontology. The funders had no role in the preparation of the manuscript.

## Ethics Statement

This case report was prepared in accordance with the Declaration of Helsinki. Ethical approval was not required for this single‐patient case report in accordance with institutional policy at the National Center for Geriatrics and Gerontology.

## Consent

Written informed consent for publication was obtained from the patient.

## Conflicts of Interest

The authors declare no conflicts of interest.

## Data Availability

The data supporting the findings of this case report are not publicly available due to patient privacy considerations.
